# Supplementary material for: Thermosensitive PBP2a requires extracellular folding factors PrsA and HtrA1 for Staphylococcus aureus MRSA β-lactam resistance
Source: Commun Biol. 2019 Nov 15;2:417. doi: 10.1038/s42003-019-0667-0 (PMC6858329; doi:10.1038/s42003-019-0667-0)
Supplement: Supplementary file 1 — Supplementary Information [file 42003_2019_667_MOESM1_ESM.pdf]

Supplementary Figures

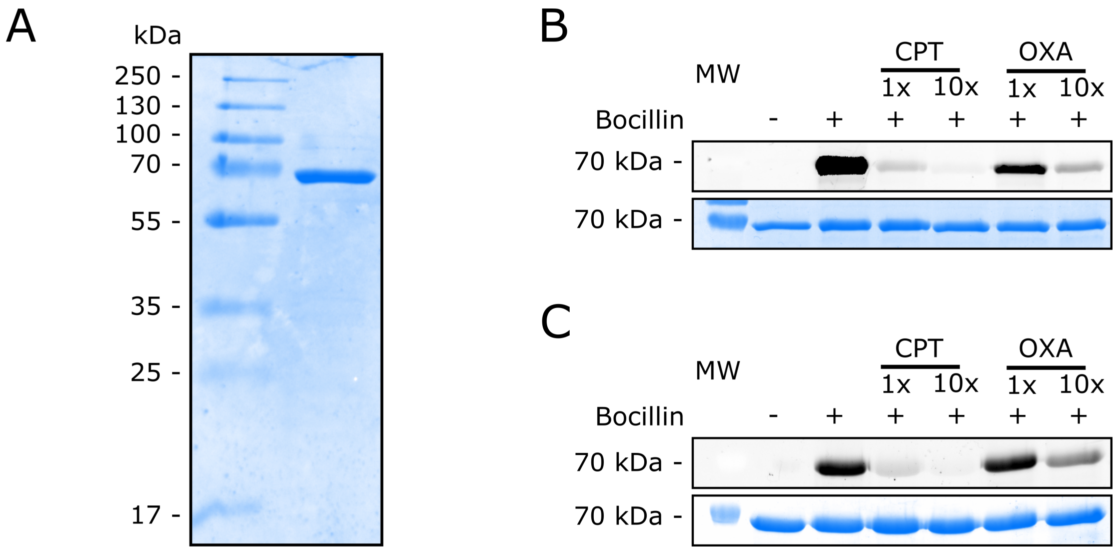

Supplementary Figure 1. **A.** Purification of PBP2A. 12% SDS PAGE gel stained with Coomassie Blue showing the purity of 5μg of the C-terminal HIS-tagged protein. **B**. Bocillin FL active site labeling and allosteric competition assay (Methods). PBP2a correct folding was judged by its ability to be deriviatized by bocillin, and subject to allosteric competition with the anti-MRSA cephalosporin β-lactam ceftaroline (CPT), but not oxacillin (OXA). The bocillin fluorescence is shown above the same gel stained with Commassie Brilliant Blue. **C**. Duplicate assay performed six months after the assay shown in panel B and using the same aliquot of purified PBP2a that been stored at 4°C, never frozen/thawed, and which did not aggregate and precipitate. This indicates that purified PBP2a is stable under these storage conditions and retains biological activity. Uncut gels of Supplementary Figure 1 are shown in Supplementary Figure 2.

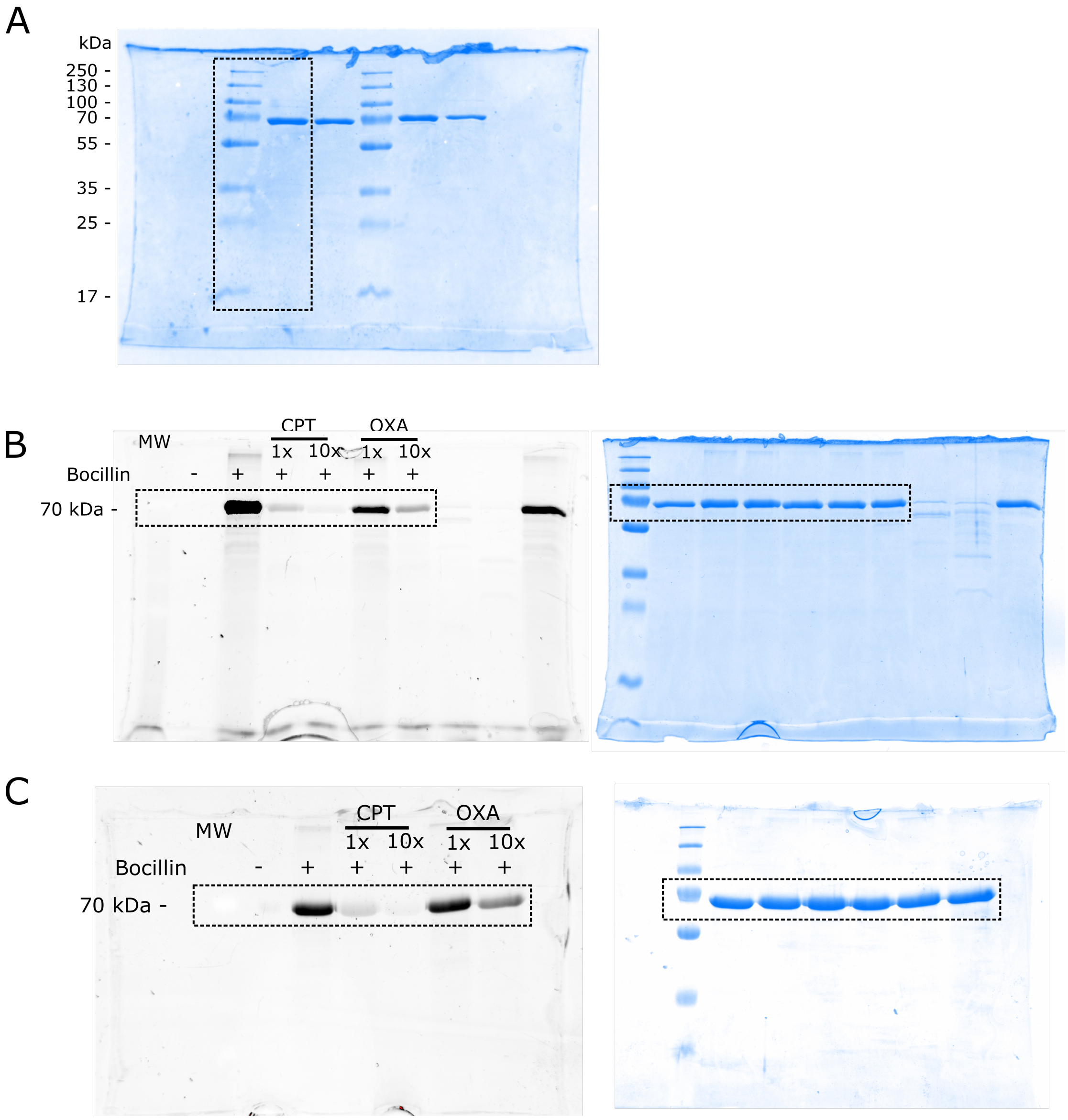

Supplementary Figure 2. Uncut gels of Supplementary Figure 1.

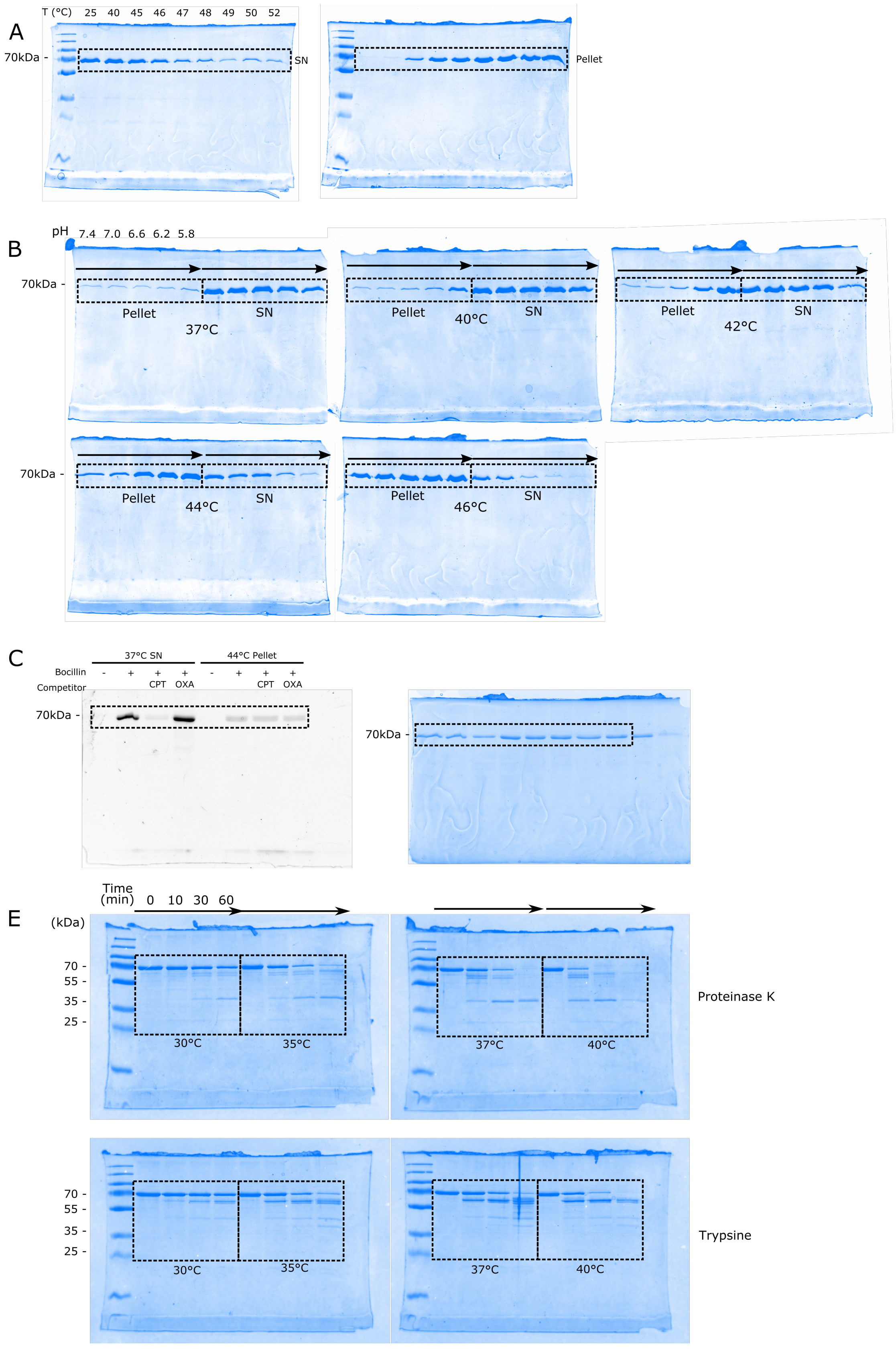

Supplementary Figure 3. Uncut gels of Figure 1.

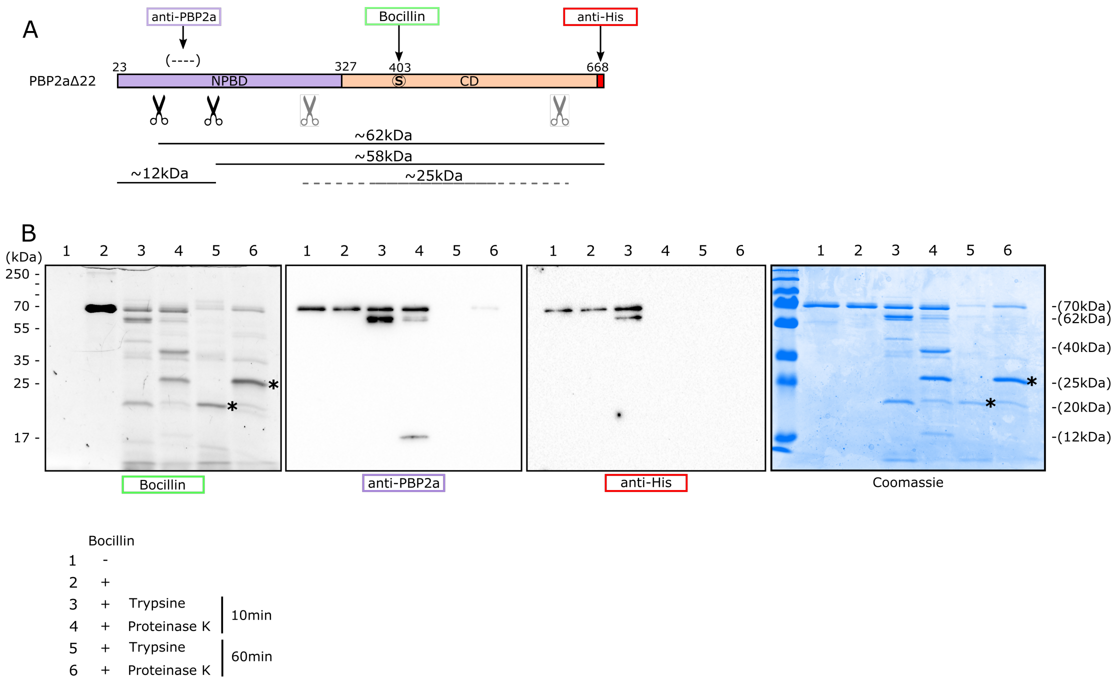

Supplementary Figure 4. **A**: The approximate molecular weights of various prominent PBP2a fragments detected by bocillin labeling/proteolysis and western analysis is shown schematically. **B**. Bocillin labeling, partial proteolysis, and western blot analysis of PBP2a. PBP2a was derivatized with bocillin FL (lanes 2-6) or not (control lane 1) and then subjected to partial proteolysis using trypsin (lanes 3, 5) or proteinase K (lanes 4, 6) for 10 minutes (lanes 3,4) or 60 minutes (lanes 5, 6). Reactions were stopped by the addition of an equal volume of 15% trichloroacetic acid, precipitated, washed in 70% ethanol, and loaded on 12% SDS PAGE gels (Methods). Bocillin labeling was detected by ImageDoc (BioRad) using Alexa 488 presets; the gel was subsequently stained with Coomassie Brilliant Blue. Aliquots from the same reactions were identically run on replicate gels and semi-dry transferred to nitrocellulose membranes for western blot analysis using either HRP-conjugated anti 6xHIS, or monoclonal anti-PBP2a. The domain architecture of PBP2a is schematized and the positions of important features: the active site serine 403 where bocillin covalently acylates, the N-terminal region where anti-PBP2a monoclonal antibody binds (the actual epitope site is proprietary by bioMérieux), the C-terminal 6xHIS tag. The extreme-C terminal 6xHis epitope was used to deduce N-terminal cleavages; the N-terminally located anti-PBP2a epitope was used to monitor the presence of fragments containing this epitope. The deduced detected fragment positions are shown with the schematic. Note the relative resistance of the catalytic domain (monitored by bocillin covalently linked at

serine 403) to prolonged proteolysis compared with the proteolytic sensitivity of the N-terminal non-penicillin binding domain (nPBD). The approximate molecular weight of the protease resistant catalytic domain: 25kDa (proteinase K) 20 kDa (trypsin).

A

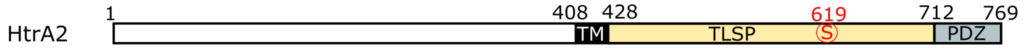

B

COL Oxacillin MICs (mg.L^{-1})	30°C	35°C	37°C	40°C
WT	>512 (512 - >512)	512	256	128 (64 - 128)
Δ*htrA2*	>512	512	256	64 (64 - 128)
Δ*prsA* Δ*htrA2*	512 (512 ->512)	256	128 (64 - 256)	16
Δ*htrA1* Δ*htrA2*	>512	512	128 (128 - 256)	32 (32 - 64)
Δ*prsA* Δ*htrA1* Δ*htrA2*	512 (256 - 512)	128 (128-256)	32 (16 - 64)	4 (4 - 8)

C

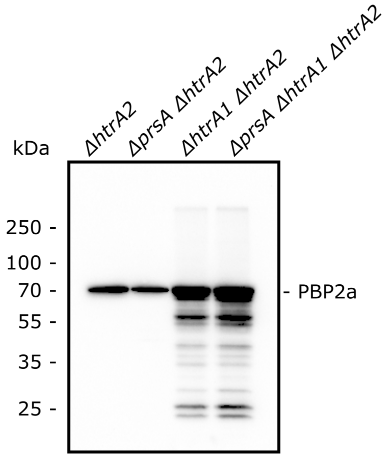

Supplementary Figure 5. A. Schematic diagram of HtrA2 domain organization showing the central transmembrane domain (TM), trypsin-like serine protease domain (TLSP), and the C-terminal PDZ domain. The active site serine of the catalytic triad is indicated. The large N-terminal domain is of unknown function. **B**. Microdilution minimal inhibitory concentration (MIC) determinations for oxacillin in MRSA strain COL derivatives with disruptions in *htrA2*. MICs are reported as modal values in mg L^{-1} with the range in parenthesis. Data are from n=3 independent biological determinations. Disruption of *htrA2* alone does not significantly alter the MIC compared with its isogenic COL parent (Table 1), nor does *htrA2* disruption significantly contribute when paired with either single (*prsA*, *htrA1*) or double (*prsA/htrA1*) mutant. All comparator strains are reported in manuscript Table 1. **C**. Western blot of membrane extracts

from the indicated cells using anti-PBP2A antibody. The disruption of *htrA2* has no measurable effect on degradation products.

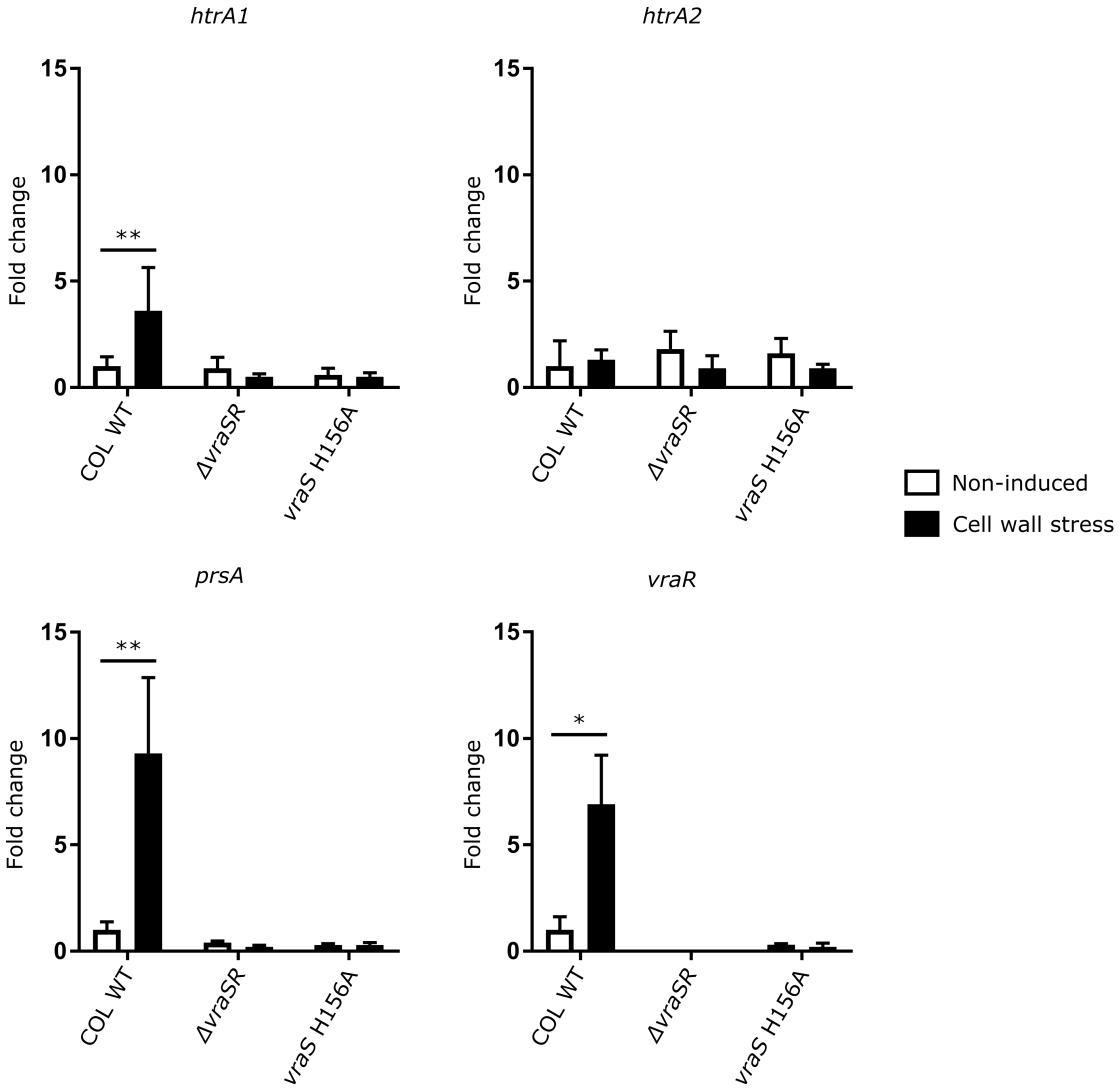

Supplementary Figure 6. qRT-PCR assay of various COL strain transcripts during exponential phase growth of the indicated strains. The same RNA preparations were used for analysis with probes specific for *htrA1*, *htrA2*, *prsA*, and *vraR* **(Supplementary Table 2***)*. Non-induced cultures were used (*open bars*) compared with cultures exposed to cell wall stress (*black bars*) using supra-MIC vancomycin 10 μg mL^{-1} for 10 min. The data is compiled from n=3 independent biological determinations. Normalizations were to 16S rRNA. Note the coordinate induction of *prsA*, *htrA1*, and *vraR* consistent with their detection in the cell wall stress regulon[30,31]. Note: *htrA2* is not induced by cell wall stress. For convenience, fold-change is reported, but all statistical analysis was performed using the raw normalized cycle threshold values. Error bars show ±SEM. * = $p<0.05$; ** $p<0.005$ by Student's two-tailed t test. The computed *P* values and data used for this determination are shown in Supplementary Table 1.

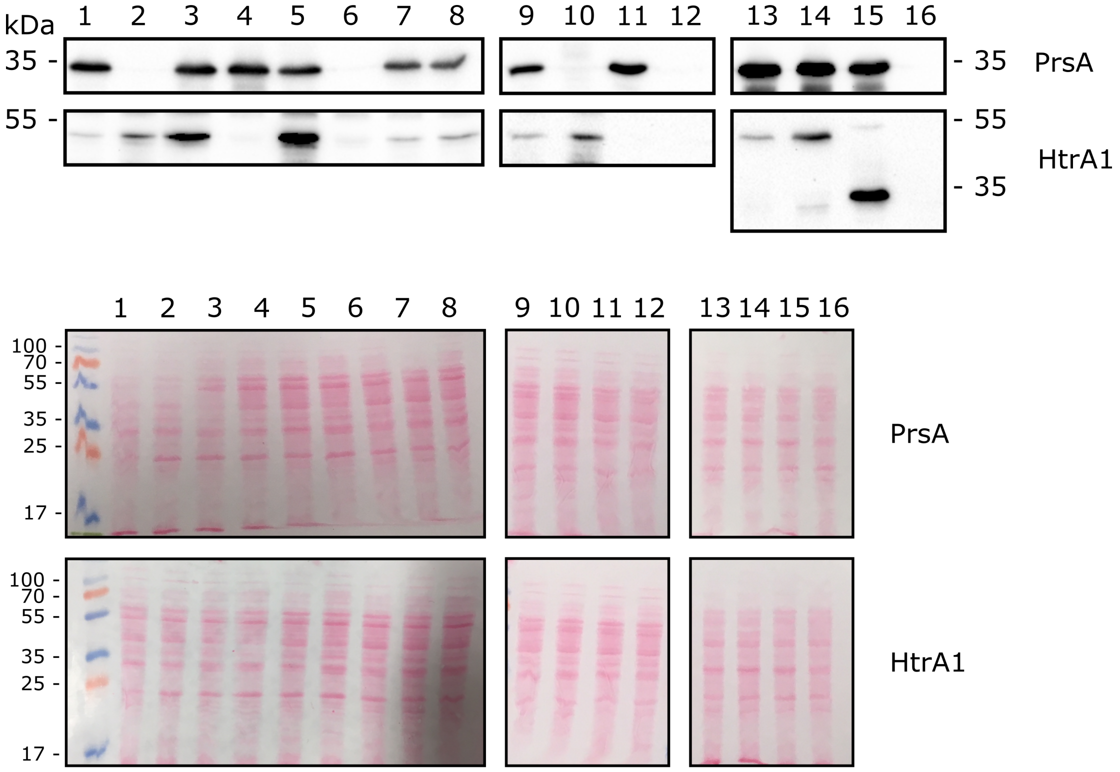

1 COL WT
2 *ΔprsA*
3 *ΔprsA-C*
4 *ΔhtrA1*
5 *ΔhtrA1-C*
6 *ΔprsAΔhtrA1*
7 *ΔVraSR*
8 VraS H156A
9 *ΔhtrA2*
10 *ΔhtrA2 ΔprsA*
11 *ΔhtrA2 ΔhtrA1*
12 *ΔhtrA2 ΔprsA ΔhtrA1*
13 *ΔprsA ΔhtrA1 pCN47:prs*A^{+},*htrA1*$^{+}$
14 *ΔprsA ΔhtrA1 pCN47:prs*A^{+}, *htrA1*-S255A
15 *ΔprsA ΔhtrA1 pCN47:prs*A^{+}, *htrA1*-ΔPDZ
16 *ΔprsA ΔhtrA1* pCN47

Supplementary Figure 7. Strain verifications by western blot analysis. Strains disrupted for *prsA*, or *htrA1*, as well as deletion strains complemented by allelic exchange, or multicopy pCN47 plasmid constructs used in the study are shown. Western blots used rabbit polyclonal anti-PrsA or rabbit custom anti-peptide anti-HtrA1 (Eurogentec, Belgium). All gels were stained with Ponceau Red for loading control verification and semi-dry transfer blot uniformity. Numbered lanes correspond to the relevant strain designations shown below.

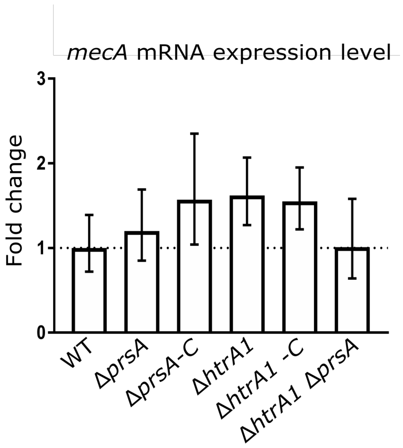

Supplementary Figure 8. Quantitative RT-PCR assay of *mecA* mRNA levels from COL wild type, or its mutant derivative strains, during exponential phase. For convenience, fold-change is plotted, but all statistical analysis was applied to the raw normalized C*t* values. Bars display ±SEM from n=3 independent biological determinations.

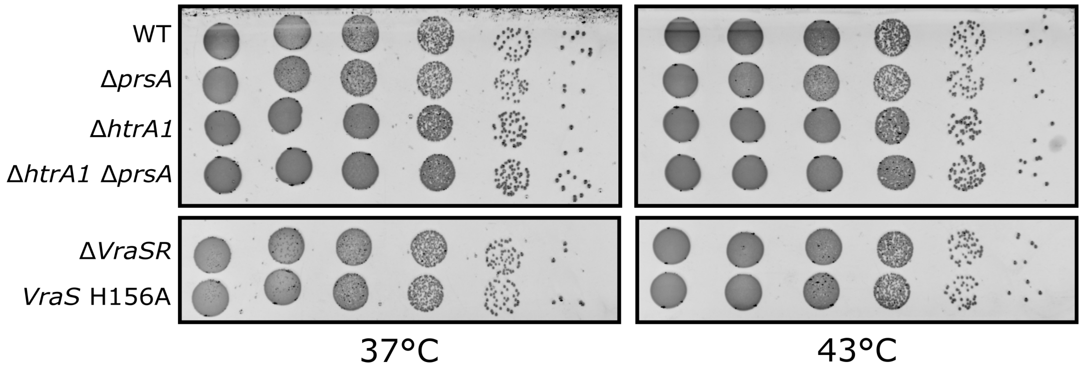

Supplementary Figure 9. Colony viability assay on MH agar plates without antibiotic. Cell cultures of the indicated strains were 10-fold serially diluted and aliquots (10 µL) spotted on agar plates and incubated at the indicated temperatures. Note that no strains are temperature sensitive at 43°C.

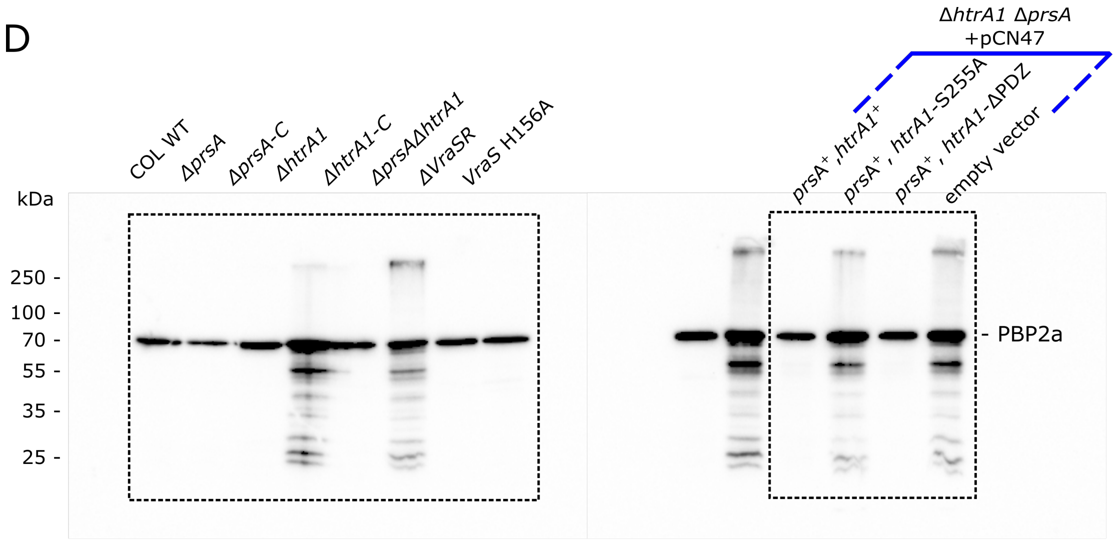

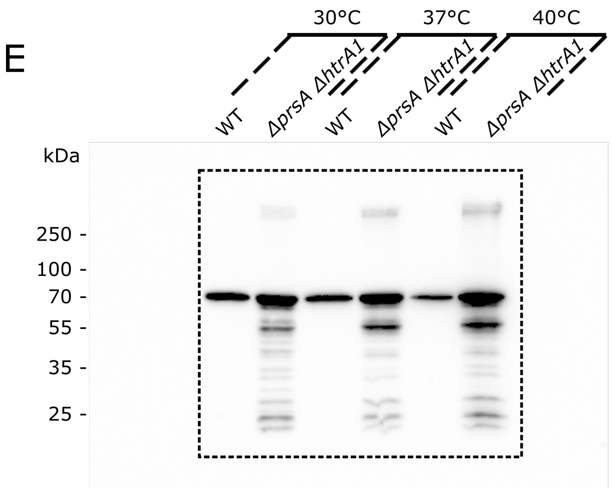

Supplementary Figure 10. Uncut membranes of Figure 2D and 2E.

Supplementary Tables

Supplementary Table 1. Raw *C*t values used for qRT-PCR analysis.

		htrA1						*p value*
		16S			*htrA1*			
		Exp 1	Exp 2	Exp 3	Exp 1	Exp 2	Exp 3	
Non-induced	WT	13.85	15.64	13.22	23.80	24.03	23.23	
	Δ*vraSR*	13.84	15.88	13.03	24.12	24.14	23.34	
	vraS H156A	13.63	15.10	13.12	24.23	24.12	24.05	
Cell wall stress	WT	13.73	15.00	13.15	21.93	21.32	21.41	0.0030
	Δ*vraSR*	13.94	14.43	13.36	24.65	24.14	24.46	0.0982
	vraS H156A	13.98	14.53	13.03	24.68	24.08	24.14	0.1725

		htrA2						*p value*
		16S			*htrA2*			
		Exp 1	Exp 2	Exp 3	Exp 1	Exp 2	Exp 3	
Non-induced	WT	13.90	14.73	13.49	31.20	28.09	28.95	
	Δ*vraSR*	13.57	14.28	13.41	29.08	27.82	27.97	
	vraS H156A	13.95	13.78	13.85	29.70	27.70	28.31	
Cell wall stress	WT	13.96	13.46	13.06	29.86	27.70	28.02	0.6592
	Δ*vraSR*	13.79	13.61	13.10	29.60	27.78	29.46	0.1836
	vraS H156A	13.59	13.28	12.99	29.49	28.24	28.74	0.1399

		prsA						*p value*
		16S			*prsA*			
		Exp 1	Exp 2	Exp 3	Exp 1	Exp 2	Exp 3	
Non-induced	WT	16.19	14.01	14.63	22.53	21.96	21.97	
	Δ*vraSR*	14.76	13.70	14.94	23.50	23.17	22.89	
	vraS H156A	14.87	14.57	14.16	23.63	24.20	22.84	
Cell wall stress	WT	15.60	14.04	14.03	19.00	18.95	17.69	0.0052
	Δ*vraSR*	14.18	13.90	13.77	23.61	23.96	22.53	0.0075
	vraS H156A	14.49	13.85	13.87	23.11	23.38	22.28	0.0839

		vraR						*p value*
		16S			*htrA2*			
		Exp 1	Exp 2	Exp 3	Exp 1	Exp 2	Exp 3	
Non-induced	WT	17.01	14.62	14.33	25.22	24.13	24.96	
	Δ*vraSR*							
	vraS H156A	15.18	14.18	13.44	26.01	25.29	25.37	
Cell wall stress	WT	15.98	13.89	13.66	21.83	21.12	20.58	0.0265
	Δ*vraSR*							
	vraS H156A	16.63	13.71	13.40	26.73	25.84	25.85	0.6551

		mecA						*p value*
		16S			*mecA*			
		Exp 1	Exp 2	Exp 3	Exp 1	Exp 2	Exp 3	
COL	WT	18.77	19.99	19.23	17.33	20.99	19.06	
	ΔprsA	18.08	20.01	19.27	16.59	20.13	19.15	0.4257
	ΔprsA -C	18.30	20.17	19.21	17.15	20.24	18.32	0.3563
	ΔhtrA1	17.98	19.89	19.35	17.67	20.38	18.72	0.9257
	ΔhtrA1 -C	17.76	19.97	19.16	17.15	20.26	18.64	0.8829
	ΔhtrA1 ΔprsA	18.40	20.08	18.94	16.37	20.42	18.80	0.2062

Supplementary Table 2. Bacterial strains and plasmids.

**Strains**	Relevant characteristics	Reference(s)
DH5α	Laboratory cloning strain, *E. coli* K-12	Gibco/BRL
Tuner (λDE3)	IPTG-inducible T/ polymerase lysogen	Novagen
ATCC29213	*S. aureus* antibiotic susceptibility reference strain	ATCC
RN4220	*S. aureus* r-m+ that accepts foreign DNA	Kreiswirth, 1983[1]
RN6390	*S. aureus* MSSA	Novick, 1990[2]; Peng, 1988[3]
Δ*htrA1*::*cat*	RN6390 Δ*htrA1*::*cat*	Rigouley, 2005[4]
Δ*htrA2*::*spc*	RN6390 Δ*htrA2*::*spc*	Rigouley, 2005[4]
COL	AJ717, HA-MRSA, plasmid cured tet^{s} Δ*bla*Δ*mecR1*Δ*mecI*Δ*MecR2*	Jousselin, 2015[5]
AJ728	COL Δ*prsA*::*kanA*	Jousselin, 2015[5]
AJ760	COL Δ*prsA*-c (complemented)	Jousselin, 2015[5]
AJ843	COL Δ*prsA*::*kanA, geh::* pCL25*prsA*-NC *tet* (Δparvulin domain)	Jousselin, 2015[5]
MR10	COL Δ*htrA1*::*cam*	This work
MR11	COL Δ*htrA2*::*spc*	This work
MR12	COL Δ*htrA1*::*cam*, Δ*htrA2*::*spc*	This work
RS373	COL *htrA1*+ (complemented MR10) kanA nearby	This work
MR13	COL Δ*prsA::kanA*, Δ*htrA1::cam*	This work
MR14	COL Δ*prsA::kanA*, Δ*htrA2::spc*	This work
MR15	COL Δ*prsA::kanA*, Δ*htrA1::cam*, Δ*htrA2::spc*	This work
MR17	COL Δ*vraRS* (related to AR868, but tet^{s})	This work; Galbusera, 2011[6]
MR18	COL *vraS* H156A (related to AR916, but tet^{s})	This work; Jousselin, 2012[7]
MR317	COL ΔPrsA, ΔhtrA1 / pCN47	This work
MR351	COL ΔPrsA, ΔhtrA1 / pCN47 *prsA+htrA1+*	This work
MR427	COL ΔprsA, ΔhtrA1 / pCN47 *prsA+*, *htrA1* H255A	This work
MR428	COL Δ*prsA*, Δ*htrA1*/ pCN47 *prsA+*, *htrA1* ΔPDZ	This work
FS241	MC4100 Δ*dnaJ*, Δ*dnaK*, Δ*tig*	Genevaux 2004[8], Angles 2017[9]
MC4100	*Δ(argF-lac)U169 araD139 rpsl150 deoC1 relA1 ptsF25 flbB5501 rbsR*	Casadaban 1976[10]
MR206	FS241 / pSE280ΔNco	Genevaux 2004[8], Angles 2017[9]
MR209	FS241 / pSE380ΔNco - Tig	Genevaux 2004[8], Angles 2017[9]
MR212	FS241 / pSE380ΔNco - TigRBD - ΔspPrsA	This work
MR216	FS241 / pSE380ΔNco - TigRBD - ΔspPrsAΔPPIase	This work
MR220	FS241 / pSE380ΔNco - ΔspPrsA	This work
**Plasmids**		
pBT2	*E.coli*/*S.aureus* shuttle vector, pC194ts	Bruckner, 1997[11]
pBT2-Kan nearby *serA*	targeting vector nearby *htrA1*	This work
pCN47	*E.coli*/*S. aureus* shuttle vector pTI181 ori, erm	Charpentier, 2004[12]
pSE380	IPTG-inducible *E.coli* expression vector	InVitrogen
pSE380DNco	pSE380ΔNco (Δ vector promoter sequence 268-306)	Genevaux 2004[8], Angles 2017[9]
pSE380DNco-tig	pSE380ΔNco - Tig	Genevaux 2004[8]
pWKGtig-PrsA	pSE380ΔNco - TigRBD - ΔspPrsA	This work
pWKGtigPrsAΔPPIase	pSE380ΔNco - TigRBD - ΔspPrsAΔPPIase	This work

pMR235	DH5α pSE380ΔNco - ΔspPrsA	This work
pMR150	DH5α pCN47 *prsA*+,*htrA*+	This work
pMR410	DH5α pCN47 *prsA*+,*htrA*S255A	This work
pMR411	DH5α pCN47 *prsA*+,*htrA*ΔPDZ	This work
pMR343	RN4220 pCN47 prsA+,htrA+	This work
pMR425	RN4220 pCN47 prsA+,htrAS255A	This work
pMR426	RN4220 pCN47 prsA+,htrAΔPDZ	This work
pET24d	T7 promoter inducible expression vector	Novagen
pET24d-CNH	COL *mecA* (PBP2A) cloned as Nco-HindIII in pET24d/C-terminal 6xHIS	This work

Supplementary Table 3. Oligonucleotide primers and probes.

Name	Sequence
**Dual operon PrsA-HtrA1 for pCN47**	
HtrA1BglIIPstREV	5'-caaactgcagagatctttatctaaagaaatctctatcgc-3'
HtrA1S255Atop	5'-attaacccaggtaacgcaggtggcgcattag-3'
HtrA1S255A bot	5'-ctaatgcgccacctgcgttacctgggttaat-3'
ΔPDZ-R306STOPPstB2REVPCR	5'-caaactgcagagatctttagcggtcaattttaccatgttttac-3'
HtrA1KpnoutG83G top	5'-caaagcaagaaattcggtaaccgttcatgaaatga-3'
HtrA1KpnoutG83G bot	5'-tcatttcatgaacggttccgaatttcttgctttg-3'
Kpn-RBS-prsA-FWD	5'-ggggtaccaaggaggaacacatatgaagatgataaacaaattaatcg-3'
overlap-RBS-htrA1-FWD	5'-ggacaatccggcatgagccaataatgaccaaggaggaacacatatgtcagattttaatcatacagatc-3'
prsAend-overlap-REV	5'-ttattggctcatgccggattgtcc-3'
**TIG-PrsA hybrids**	
EcTigEcoFWD	5'-cggaattcatgcaagtttcagttgaaacc-3'
TIG148-PrsAΔ21-bottom	5'-ctttagagtctgtggcactagcgccctgctgtttacgcagagtatccag-3'
TIG148-PrsAΔ21-top	5'-ctggatactctgcgtaaacagcagggcgctagtgccacagactctaaag-3'
SaPrsAXbaREV	5'-gctctagattattggctcatgccggattgtcc-3'
SaPrsABamΔ21MET	5'-gcggatccatgggcgctagtgccacagactctaaag-3'
**PBP2A cloning and expression in pET24d**	
CHisMecAFNco	5'-actaccatggcttcaaaagataaagaaattaata-3'
mecARHindIII	5'-atttaagcttttcatctatatcgtattttttattacc-3'
**Strain verifications**	
Cam-1	5'-gtggctctaacttatcccaataacc-3'
Cam-2	5'-caggattgtttatgaactctattcagg-3'
ErmB-1	5'-ccaatttcgtaaacggtatcgg-3'
ErmB-2	5'-ctatatacgtactttgtttcaaaatggg-3'
PBP2AEcoFWD	5'-cggaattcatgaaaaagataaaaattgttccac-3'
PBP2ABamREV	5'-cgggatccttattcatctatatcgtattttttattacc-3'
COLccrAKpnFWD	5'-cggggtaccatgaaacaagcaataggttacttacg-3'
COLccrABamREV	5'-cgggatccttatgcaatcgatgattgcatggtttg-3'
OL Kan1-100nest	5'-aatatggcgcttcatagagtaattc-3'
OL Kan2-89nest	5'-ggcgttgacaggtatgagactacaa-3'
seqMecA1F	5'-cacttggtatatcttcaccaacacc-3'
seqMecA2F	5'-agcatcaatagttagttgaat-3'
T7Fwd	5'-taatacgactcactatagg-3'
T7term	5'-tagttattgctcagcggtgg-3'
OL1.htrA1 F downstream cds	5'-tcactgaatacattgtgcca-3'
OL2.htrA1 Rev WT not mut	5'-caaattgtaatcctaatggg-3'
OL3.htrA1 F WT not mut	5'-ggtatcatttcagcaagcgaacg-3'
OL4.htrA1 Rev upstream cds	5'-ggtatcatttcagcaagcgaacg-3'

OL5.htrA1 Rev cat mut not WT	5'-cgattttgtcctgtctgtgc-3'
OL6.htrA1 Fwd cat mut not WT	5'-gacgttgagcctcggaaccc-3'
OL7.htrA1 F cds	5'-gcagtaactgatattgctgtactta-3'
OL8.htrA1 Rev cds	5'-caactaaattaccattaatatctac-3'
OL9.htrA2 F downstream cds	5'-attgaatggagtggacattag-3'
OL10.htrA2 Rev WT not mut	5'-catcacttatcgatggtgtat-3'
OL11.htrA2 F WT not mut	5'-gcaacttcttcagacagttca-3'
OL12.htrA2 Rev upstream cds	5'-gacacgttgttcacctcaac-3'
OL13.htrA2 Rev spc mut not WT	5'-gagtcaaaaggatctaggtga-3'
OL14.htrA2 F spc mut not WT	5'-agtgaaacaatttgtcccac-3'
**KanA insertion nearby HtrA1 downstream of *serA* for strain *htrA1*-c**	
OL17-KpnFWD-SA1545	5'-gacataggtaccgggcagctatcgatgtgttt-3'
OL18-BAMIg1545-1546	5'-gttccaggatccaaaatgccaagaagcaatgg-3'
OL19-BAMIg1545-1546FWD	5'-gaccacggatccttcattattaatttcattgtaactc-3'
OL 20-XbaREV-SA1546	5'-gtcatgtctagagcctgtcgaattgaagttgc-3'
**qRTPCR**	
qRT-PCR mecA	
mecA 1038 F	5'- aggtactgctatccaccctcaaac-3'
mecA 1134 R	5'- ttcgttactcatgccatacataaatg-3'
mecA 1069	5' 6-FAM - ttattagcacttgtaagcacaccttca - MGB Eclipse 3'
qRT-PCR htrA1	
htrA1 659F	5'-ctggtatcatttcagcaagcga-3'
htrA1 740R	5'-tctgtttgaagaacgctaacttttgt-3'
htrA1 682T	5'-cgtacgattgacgctgagacaactggtg-3'
qRT-PCR htrA2	
htrA2 190F	5'-aaaaatagtttatcatcgcatttagaaca-3'
htrA2 229R	5'-tgttatatgctgacttactatcctgattacttt-3'
htrA2 220T	5'-agaaaccgtgatgttaatgagaataaagctgaagaaagt-3'
qRT-PCR prsA	
prsA-F	5'-agttaatgataagaagattgacga-3'
prsA-R	5'-gaagggccttttcaaatttatcttt-3'
prsA-P	5'-tgaaaaaatgcaaaagcaatacggcgg-3'
qRT-PCR VraRST	
vraR-F	5'-tgcttacagaacgagaaatggaaa-3'
vraR-R	5'-ccgttttaatagtaatatgcgatgca-3'
vraR-P	5'-tgattgcgaaaggttactcaaatcaagaaat-3'
qRT-PCR 16S	
16S-F	5'-gatagagccttccccttcgg-3'
16S-R	5'-ccggcagtcaacttagagtgc-3'
16S-P	5'-acatctcacgacacgagctgacgaca-3'

Supplementary References

1 Kreiswirth, B. N., Lofdahl, S., Betley, M. J., O'Reilly, M., Schlievert, P. M. *et al.* The toxic shock syndrome exotoxin structural gene is not detectably transmitted by a prophage. *Nature* **305**, 709-712, doi:10.1038/305709a0 (1983).

2 Novick, R. P. in *Molecular Biology Of the Staphylococci* (ed R. P. Novick) 1-40 (VCH Publishing, 1990).

3 Peng, H. L., Novick, R. P., Kreiswirth, B., Kornblum, J. & Schlievert, P. Cloning, characterization, and sequencing of an accessory gene regulator (agr) in Staphylococcus aureus. *Journal of bacteriology* **170**, 4365-4372, doi:10.1128/jb.170.9.4365-4372.1988 (1988).

4 Rigoulay, C., Entenza, J. M., Halpern, D., Widmer, E., Moreillon, P. *et al.* Comparative analysis of the roles of HtrA-like surface proteases in two virulent Staphylococcus aureus strains. *Infection and immunity* **73**, 563-572, doi:10.1128/iai.73.1.563-572.2005 (2005).

5 Jousselin, A., Manzano, C., Biette, A., Reed, P., Pinho, M. G. *et al.* The *Staphylococcus aureus* Chaperone PrsA Is a New Auxiliary Factor of Oxacillin Resistance Affecting Penicillin-Binding Protein 2A. *Antimicrobial agents and chemotherapy* **60**, 1656-1666, doi:10.1128/aac.02333-15 (2015).

6 Galbusera, E., Renzoni, A., Andrey, D. O., Monod, A., Barras, C. *et al.* Site-specific mutation of *Staphylococcus aureus* VraS reveals a crucial role for the VraR-VraS sensor in the emergence of glycopeptide resistance. *Antimicrobial agents and chemotherapy* **55**, 1008-1020, doi:10.1128/aac.00720-10 (2011).

7 Jousselin, A., Renzoni, A., Andrey, D. O., Monod, A., Lew, D. P. *et al.* The posttranslocational chaperone lipoprotein PrsA is involved in both glycopeptide and oxacillin resistance in *Staphylococcus aureus*. *Antimicrobial agents and chemotherapy* **56**, 3629-3640, doi:10.1128/aac.06264-11 (2012).

8 Genevaux, P., Keppel, F., Schwager, F., Langendijk-Genevaux, P. S., Hartl, F. U. *et al.* In vivo analysis of the overlapping functions of DnaK and trigger factor. *EMBO reports* **5**, 195-200, doi:10.1038/sj.embor.7400067 (2004).

9 Angles, F., Castanie-Cornet, M. P., Slama, N., Dinclaux, M., Cirinesi, A. M. *et al.* Multilevel interaction of the DnaK/DnaJ(HSP70/HSP40) stress-responsive chaperone machine with the central metabolism. *Scientific reports* **7**, 41341, doi:10.1038/srep41341 (2017).

10 Casadaban, M. J. Transposition and fusion of the lac genes to selected promoters in Escherichia coli using bacteriophage lambda and Mu. *Journal of molecular biology* **104**, 541-555 (1976).

11 Bruckner, R. Gene replacement in Staphylococcus carnosus and Staphylococcus xylosus. *FEMS microbiology letters* **151**, 1-8, doi:10.1111/j.1574-6968.1997.tb10387.x (1997).

12 Charpentier, E., Anton, A. I., Barry, P., Alfonso, B., Fang, Y. *et al.* Novel cassette-based shuttle vector system for gram-positive bacteria. *Applied and environmental microbiology* **70**, 6076-6085, doi:10.1128/aem.70.10.6076-6085.2004 (2004).
