# Supplementary material for: Thermosensitive PBP2a requires extracellular folding factors PrsA and HtrA1 for Staphylococcus aureus MRSA β-lactam resistance
Source: Commun Biol. 2019 Nov 15;2:417. doi: 10.1038/s42003-019-0667-0 (PMC6858329; doi:10.1038/s42003-019-0667-0)
Supplement: Supplementary file 2 — Description of Additional Supplementary Files [file 42003_2019_667_MOESM2_ESM.docx]

**Supplementary Data 1** contains the source data for differential scanning fluorimetry shown in Figure 1D in Excel format.

The file contains the arbitrary fluorescence units versus temperature recorded in half degree °C increments. The sections are delimited by pH (7.4 or 5.8) and the independent biological experiments by assay date. Blank (lacking PBP2a, buffer and Sypro orange only) or experimental (with PBP2a) are supplied.
