# Supplementary material for: Thermosensitive PBP2a requires extracellular folding factors PrsA and HtrA1 for Staphylococcus aureus MRSA β-lactam resistance
Source: Commun Biol. 2019 Nov 15;2:417. doi: 10.1038/s42003-019-0667-0 (PMC6858329; doi:10.1038/s42003-019-0667-0)
Supplement: Supplementary file 5 — Reporting Summary [file 42003_2019_667_MOESM5_ESM.pdf]

natureresearch

Corresponding author(s): KELLEY, William

Last updated by author(s): October 14, 2019

Reporting Summary

Nature Research wishes to improve the reproducibility of the work that we publish. This form provides structure for consistency and transparency in reporting. For further information on Nature Research policies, see Authors & Referees and the Editorial Policy Checklist.

Statistics

For all statistical analyses, confirm that the following items are present in the figure legend, table legend, main text, or Methods section.

n/a	Confirmed	
☐	☒	The exact sample size (*n*) for each experimental group/condition, given as a discrete number and unit of measurement
☐	☒	A statement on whether measurements were taken from distinct samples or whether the same sample was measured repeatedly
☐	☒	The statistical test(s) used AND whether they are one- or two-sided<br>*Only common tests should be described solely by name; describe more complex techniques in the Methods section.*
☒	☐	A description of all covariates tested
☒	☐	A description of any assumptions or corrections, such as tests of normality and adjustment for multiple comparisons
☐	☒	A full description of the statistical parameters including central tendency (e.g. means) or other basic estimates (e.g. regression coefficient) AND variation (e.g. standard deviation) or associated estimates of uncertainty (e.g. confidence intervals)
☐	☒	For null hypothesis testing, the test statistic (e.g. F, t, r) with confidence intervals, effect sizes, degrees of freedom and P value noted<br>*Give P values as exact values whenever suitable.*
☒	☐	For Bayesian analysis, information on the choice of priors and Markov chain Monte Carlo settings
☒	☐	For hierarchical and complex designs, identification of the appropriate level for tests and full reporting of outcomes
☒	☐	Estimates of effect sizes (e.g. Cohen's d, Pearson's r), indicating how they were calculated

Our web collection on statistics for biologists contains articles on many of the points above.

Software and code

Policy information about availability of computer code

Data collection	Quantitative real time PCR and fluorimetry data were acquired on a BioRad CFX detection system. Chemiluminescent western blot data and bocillin fluorescence were captured with a BioRad MP imaging system using the manufacturer's presets (bocillin FL was captured on the Alexa 488 channel).
Data analysis	Quantitative RT-PCR and fluorimetry data were exported to Excel file format and plotted and analyzed using GraphPad versions 7 and 8. Digital images were exported from the Chemidoc system as TIF files and imported in Inkscape version 0.92 for figure construction and display. The source data for differential scanning fluorimetry is provided in Supplementary Data file 1 in Excel format. Protein database (PDB) structure coordinates were downloaded and used with Chimera versions 1.12-1.5 (University of California San Francisco). Statistical analysis for the qRT-PCR experiments was performed with Excel and plotted using GraphPad 7. The data in Supplementary Figure 8 used Student's paired t test for n=3 independent determinations. The data in Supplementary Figure 6 also used Student's paired t test for n=3 determinations, but with the Bonferroni correction applied. The raw cycle threshold values used for both analyses are supplied in supplementary data file Table 1. MIC antibiotic susceptibility testing uses standard modal value reporting with the range of other measured values, if observed, indicated in parenthesis.

For manuscripts utilizing custom algorithms or software that are central to the research but not yet described in published literature, software must be made available to editors/reviewers. We strongly encourage code deposition in a community repository (e.g. GitHub). See the Nature Research guidelines for submitting code & software for further information.

Data

Policy information about availability of data

All manuscripts must include a data availability statement. This statement should provide the following information, where applicable:

- Accession codes, unique identifiers, or web links for publicly available datasets
- A list of figures that have associated raw data
- A description of any restrictions on data availability

All data used in this manuscript are included within the manuscript and its supplementary accompanying files.

Field-specific reporting

Please select the one below that is the best fit for your research. If you are not sure, read the appropriate sections before making your selection.

☒ Life sciences ☐ Behavioural & social sciences ☐ Ecological, evolutionary & environmental sciences

For a reference copy of the document with all sections, see nature.com/documents/nr-reporting-summary-flat.pdf

Life sciences study design

All studies must disclose on these points even when the disclosure is negative.

Sample size	All experiments used a minimum of three independent determinations. For MIC assays, EUCAST standards were followed (www.eucast.org) using triplicate assay of at least three independent cultures. Bacteria used for culture were obtained in each instance from single colony isolation from -80C master freezer stocks.
Data exclusions	No data were excluded. MRSA strains cultivated in the laboratory are known to have unstable SCCmec elements encoding mecA. MIC assays were assured by PCR analysis of mecA sequence and ccr (primers indicated in supplementary Table 3) to be certain of strain integrity. Although pCN47-based plasmids harboring htrA1 alone could be constructed, they could not be transformed into the appropriate S. aureus mutant strains for unknown reasons. pCN47-based plasmids were therefore constructed for dual expression of both prsA and htrA1 in a hybrid operon format to expedite complementation analysis of the double mutant strains. These plasmids worked without difficulty.
Replication	All attempts at experimental replication were successful.
Randomization	N/A
Blinding	N/A

Reporting for specific materials, systems and methods

We require information from authors about some types of materials, experimental systems and methods used in many studies. Here, indicate whether each material, system or method listed is relevant to your study. If you are not sure if a list item applies to your research, read the appropriate section before selecting a response.

Materials & experimental systems

n/a	Involved in the study	
☐	☒	Antibodies
☒	☐	Eukaryotic cell lines
☒	☐	Palaeontology
☒	☐	Animals and other organisms
☒	☐	Human research participants
☒	☐	Clinical data

Methods

n/a	Involved in the study	
☒	☐	ChIP-seq
☒	☐	Flow cytometry
☒	☐	MRI-based neuroimaging

Antibodies

Antibodies used	PBP2a was detected with the commercial monoclonal anti-PBP2a employed in the Slidex MRSA detection kit (BioMérieux reference 73117). HRP-conjugated mouse monoclonal anti-6xHIS was obtained from Invitrogen. Rabbit polyclonal anti-PrsA antibody was the kind gift of Dr. Vesa Kontinen (Helsinki, Finland). Rabbit anti-HtrA1 was custom made by Eurogentec (Belgium) against a peptide linked to KLH, as described in the methods.
Validation	The anti-PBP2a is a widely used commercial product; it was verified by absence of reactivity to S. aureus MSSA strains, which do not encode mecA, and specific detection of PBP2a in MRSA strains. The precise epitope recognized by the antibody is a BioMérieux trade secret, but is situated in the N-terminal domain as we show is SupplementaryFigure 4. Anti-6xHIS shows no background reactivity with proteins lacking the 6X HIS tag in our extracts. PBP2a with the C-terminal 6xHIS tag loses antibody

reactivity following mild proteolysis, also depicted in Supplementary Figure 4. Anti PrsA antibodies have been used and verified previously and appropriate references indicated in the legend to supplementary Figure 7. Anti-HtrA1 antibodies were verified in our laboratory using extracts from S. aureus wild type or htrA1 disruption strains. The antibody does not cross-react with HtrA2.
